# Semantic Segmentation and Effect Optimization of 3D Point Cloud Based on 2D Semantic Segmentation and Clustering for Construction Machinery Unstructured Environment

**DOI:** 10.3390/s26041257

**Published:** 2026-02-14

**Authors:** Shengjie Fu, Qipeng Cai, Zhongshen Li, Wentao Wang, Tianliang Lin, Qihuai Chen, Zhaoyuan Yao

**Affiliations:** 1 College of Mechanical Engineering and Automation, Huaqiao University, Xiamen 361021, China; xmshengjie@163.com (S.F.);; 2 Fujian Key Laboratory of Green Intelligent Drive and Transmission for Mobile Machinery, Xiamen 361021, China

**Keywords:** construction machinery, intelligentization, environment perception, multi-sensor fusion, unstructured, semantic segmentation

## Abstract

The operational environment of construction machinery is predominantly unstructured, characterized by rapid changes, high complexity, and irregularly distributed objects. This poses significant challenges for 3D semantic perception, particularly due to the high cost of acquiring point cloud semantic labels. To address this, a novel 3D semantic perception scheme is proposed for such unstructured environments. This scheme integrates image semantic segmentation results with point cloud clustering via perspective projection. The projection parameters are refined using Particle Swarm Optimization (PSO), and the semantic consistency of the fused results is further enhanced by a Kd-tree-based radius nearest neighbor (RNN) matching algorithm. Consequently, a weakly supervised framework is established that achieves accurate 3D semantic understanding using only 2D image labels, eliminating the need for annotated 3D point clouds. The feasibility and effectiveness of the scheme are validated through a dedicated unstructured scene dataset and real-world testing. Results demonstrate its capability to effectively perceive 3D semantic information and reconstruct target contours, achieving a mean Pixel Accuracy (mPA) of 84.72% and a mean Intersection over Union (mIoU) of 75.85%.

## 1. Introduction

Construction machinery plays an important role in national economic construction and emergency rescue. Intelligent and unmanned construction machinery can effectively alleviate operation difficulty caused by the harsh working environment and the threat of the dangerous situation of the rescue and disaster relief site. Digital acceleration and intelligent development have become the consensus in this industry [1].

Environmental perception is essential for the intelligent development of construction machinery. However, there are a large number of unstructured scenes for construction machinery. Additionally, the working environment is highly dynamic, and the scene is often cluttered with numerous irregularly distributed objects. This inherent complexity and variability make it difficult for a single sensor to achieve effective perception. Multi-sensor fusion has become an important trend of its development.

At present, in the aspect of multi-sensor environment semantic perception, with the development of deep learning, a large number of perception tasks based on network fusion methods have emerged [2,3]. F-PointNet projected the image 2D candidate box into the 3D space and used the 3D cone view formed by the 2D box to merge the features of the points in the 3D space as the candidate box in the 3D space. At the same time, the outlier points on the cone view were removed by instance segmentation. Finally, the network was used to predict the category information of the object, which improved the network prediction accuracy to a certain extent [4]. PointPainting mapped the semantic information of the image to the point cloud data by projection and input the semantic point cloud with semantic information into the target detection network to improve the regression performance of target detection through semantic information. However, the low mapping accuracy led to limited performance improvement [5]. The representative work of MV3D proposed by Chen et al. was based on the mapping of 3D candidate boxes. The convolutional network was used to extract features on the three inputs of the point cloud, the front view, the top view and the image, respectively. Then, the 3D candidate boxes extracted from the top view point cloud were mapped to other modes for feature extraction. Finally, based on the candidate boxes, all modes were fused and detected. The prediction accuracy was improved to a certain extent, but the fusion result depended on the generation of the initial candidate boxes [6]. The Mvx-Net network improved the dimension of fusion information by directly fusing the feature vectors on the image with the voxelized vector point features of the point cloud [7]. The CLOCs network used the output results from two different branches of point cloud and image to fuse the final output prediction results by calculating confidence, distance, and intersection ratio, but it was necessary to unify the output results of the two modes [8]. Zhuang et al. proposed the PMF network, in which the image data and the forward-looking projection data of the point cloud were input into the two-stream architecture. The residual-based fusion module of the point cloud branch could learn the complementary features of the image RGB and the point cloud data, and it obtained better semantic segmentation results [9]. The above fusion algorithms improved the accuracy of the network through the mapping of image semantics to point clouds, but they all relied on the acquisition of point cloud semantic labels, and the computational resource consumption is huge.

Most of the aforementioned fusion algorithms follow the “data-level” or “feature-level” fusion paradigms. While they improve accuracy, their common prerequisite is reliance on large-scale, high-quality point cloud semantic labels for end-to-end training, which consumes enormous computational resources. Meanwhile, to reduce annotation costs in the 2D semantic segmentation domain, weakly supervised methods such as mask-supervised learning [10] have been proposed, achieving significant progress using only image-level or bounding box labels. In 3D point cloud processing, unsupervised or self-supervised geometric feature learning and clustering represent long-standing research directions. For instance, methods like graph-regularized sparse coding [11] have been successfully applied to 3D shape representation and clustering. However, the key challenge for practical 3D semantic perception in unstructured environments lies in effectively and robustly fusing low-cost, readily available 2D semantic knowledge with unlabeled 3D geometric clustering results, thereby completely eliminating dependence on 3D point cloud semantic labels.

Unlike fully supervised fusion paradigms, this work pursues a fundamentally different direction by investigating reliable 3D semantic perception using only 2D image labels, thereby entirely bypassing the need for costly 3D point cloud annotations. This approach is especially valuable in unstructured scenarios, where 3D labels are inherently scarce. The novelty of the approach lies not in the concept of projection itself, but in the specific technical innovations introduced. These include PSO-based calibration refinement and Kd-tree-guided semantic consistency optimization. Together, they enable robust performance under the real-world conditions of weak supervision and imperfect calibration typically found on construction sites.

Currently, there is a lack of high-quality 3D semantic segmentation data sets. Additionally, in unstructured scenes, the scene exhibits high complexity with numerous irregularly distributed objects, and the shape changes rapidly, which makes the acquisition cost of point cloud semantic labels increase sharply. The environmental semantic perception technology in the field of passenger vehicles cannot be directly applied to construction machinery. In this paper, multi-sensor fusion is carried out based on camera and LiDAR. Point cloud clustering and image semantic segmentation results are fused by perspective projection. 3D semantic perception in unstructured environment is realized only by image semantic labels. Particle Swarm Optimization [12] (PSO) is used to optimize the perspective projection fusion process, and Kd-tree based radius nearest neighbor (RNN) is used to match the fusion results. Finally, a semantic data set of the excavator unstructured actual working environment is constructed for perception experiments to verify the effectiveness of the proposed scheme. Notably, the accurate 3D semantic understanding achieved by our fusion framework serves as a critical foundation for dynamic 4D scene analysis (3D + time), which is essential for advanced applications like progress monitoring and constructability review in construction projects [13,14].

Our contributions are summarized as follows: The first contribution is a weakly-supervised 3D semantic perception pipeline that operates exclusively on 2D image labels, completely bypassing the cost and effort of 3D point cloud annotation. This approach establishes a new, practical paradigm for perception in unstructured environments. The second contribution lies in our dual-branch fusion architecture and its novel optimization components: we use a PSO-based mechanism to correct weak calibration in projection and an adaptive RNN algorithm to enforce semantic consistency, directly addressing the key bottlenecks in fusion reliability. As a third contribution, competitive performance is demonstrated through a dedicated dataset and rigorous experimentation, with the framework achieving an mIoU of 75.85% and an mPA of 84.72%. A compelling balance is thus offered between high accuracy and low resource expenditure.

## 2. System Overview and Methodology

### 2.1. Preliminary Consideration

Aiming at the task requirements of construction machinery under unstructured working conditions, the 3D semantic perception task of an unstructured environment is studied and analyzed. The 3D semantic perception scheme of a construction machinery unstructured scene is designed.

#### 2.1.1. Perception Hardware Platform Design

**Figure 1:**
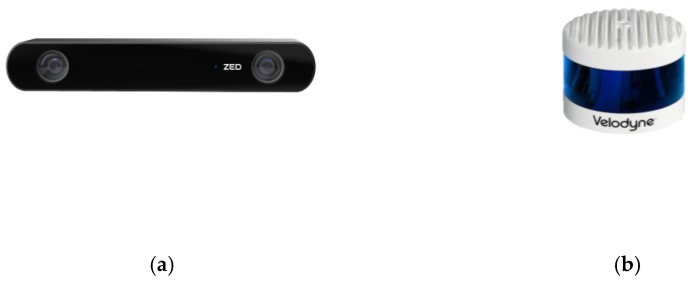
Perception sensors. (**a**) Binocular camera; (**b**) Velodyne LiDAR.

**Figure 2:**
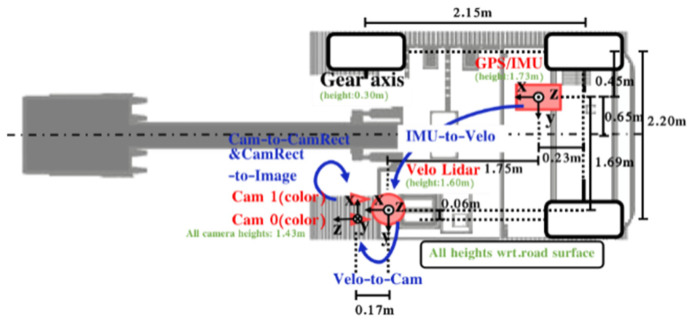
Sensor layout.

In this paper, a binocular camera and LiDAR are selected as fine-grained perception sensors, as shown in Figure 1, to achieve multi-level perception complementation. An intelligent excavator test platform is built, equipped with perception sensors as well as integrated navigation and on-board computing platforms. The sensor layout is shown in Figure 2.

The spatio-temporal synchronization processing of the forward-looking multi-sensor perception platform ensures the accurate acquisition of environmental sensing data. A robot operating system (ROS) is used for sensor information communication. NVIDIA AGX Orin (Santa Clara, CA, USA) and workstation are used as data acquisition and storage devices.

The average error, standard deviation, maximum error and minimum error results are analyzed by multiple sets of synchronous data, as shown in Table 1. The time error between the sensors at the same time will lead to a certain error in the position of the obstacle detected by the sensor. As the construction machinery moves at different speeds, the position error is also different. The excavator in this paper is calculated with the fastest moving speed of 0.51 m/s in the wor-king process. To quantify the impact of synchronization errors on real-time perception, the resulting spatial displacement errors are calculated. Based on the excavator’s maximum operating speed of 0.51 m/s and the maximum observation time error of 0.04577 s, the worst-case spatial error was calculated as 0.0234 m (0.51 m/s × 0.04577 s). Considering the large size of primary targets (e.g., mounds and construction machinery are typically meters in scale) and the fact that the excavator operates at low speeds with ample stopping distance, this error is deemed acceptable for the current perception task.

The ROS camera internal parameter calibration function package is used to calibrate the internal and external parameters of the camera. The process uses the principle of checkerboard calibration method [15]. By using the calibration board to represent the known points in the world coordinate system, the 2D coordinates of the corner points on the calibration board are captured from multiple angles. By locating the corner points on the point cloud data, the corresponding relationship between the corner points on each image in the three-dimensional space and the two-dimensional image plane is constructed. The camera imaging model principle predicts the position of the point in the 3D space to obtain the position in the 2D pixel plane, calculates the predicted position and the position error obtained by the calibration board, and uses the nonlinear two method to minimize the error to obtain the camera internal parameters and distortion coefficients. The spatial calibration result of the sensor can be obtained as shown in Table 2; the resulting re-projection error was 0.454 pixels (on a 1280 × 720 image), indicating a high-precision calibration, which provides a reliable foundation for our fusion pipeline.

#### 2.1.2. Perception Software Platform Design

To carry out the 3D perception test and data acquisition in the actual working environment, the environment configuration of the construction machinery computing platform is carried out. The specific configuration is shown in Table 3.

**Figure 3:**
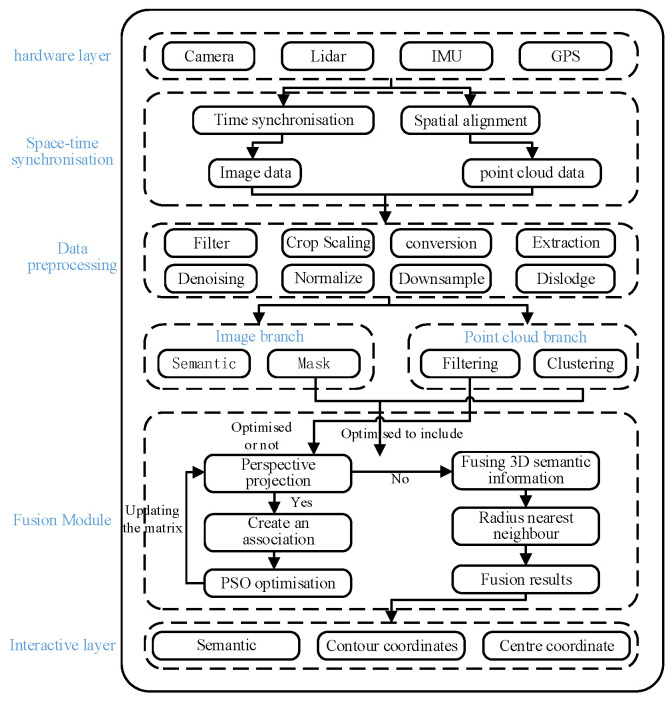
3D semantic perception scheme for unstructured environment.

A post-fusion framework with complementary and decoupling characteristics is adopted to integrate camera and LiDAR perceptions. Image semantic labels are significantly less costly to annotate than point cloud labels, and their acquisition is more mature. Supervised learning is well-established for image semantic segmentation, while unsupervised learning better exploits the rich spatial–geometric features of LiDAR point clouds. Therefore, a 3D semantic perception scheme for unstructured environments is constructed, combining a supervised image branch with an unsupervised point cloud branch. As shown in Figure 3, the scheme comprises a point cloud branch, an image branch, and a fusion module. The image segmentation and point cloud clustering results are first fused via perspective projection. They are then optimized using PSO and a Kd-tree-based Recurrent Neural Network (RNN) matching algorithm to produce the final 3D semantic perception output.

#### 2.1.3. Dataset Construction

Current public datasets predominantly feature structured urban scenes, which are inadequate for addressing the perception needs of construction machinery operating in unstructured environments. Consequently, a dedicated multi-sensor fusion semantic segmentation dataset for such unstructured scenarios must be constructed. This dataset supplies pixel-level semantic training labels for the deep learning-based image branch, alongside referenced point cloud semantic ground truth for evaluating the fused 3D semantic perception outcomes.

**Figure 4:**
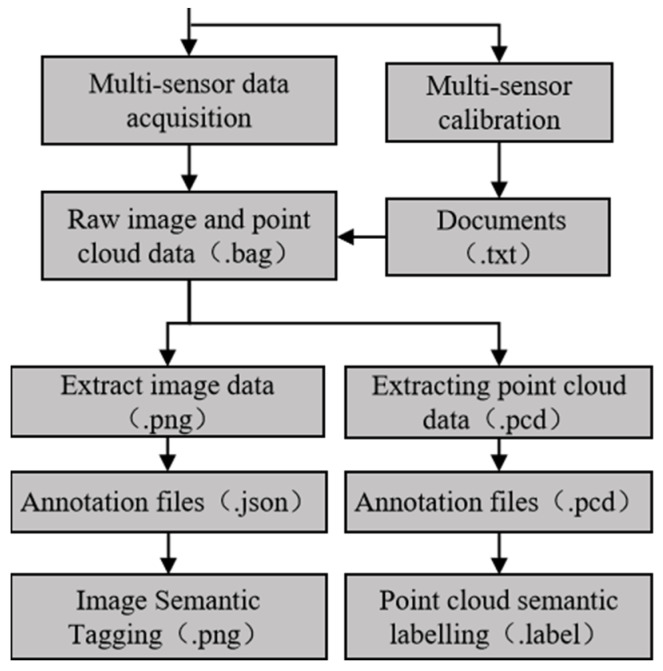
Workflow of dataset construction.

Data collection is conducted using a spatiotemporally synchronized perception platform. To minimize redundancy, frames are subsampled at a rate of one frame per ten. Adhering to the KITTI [16] dataset format, LabelMe and a point cloud labeling tool are utilized to annotate the image and point cloud data, respectively. The overall workflow for dataset construction is depicted in Figure 4.

**Figure 5:**
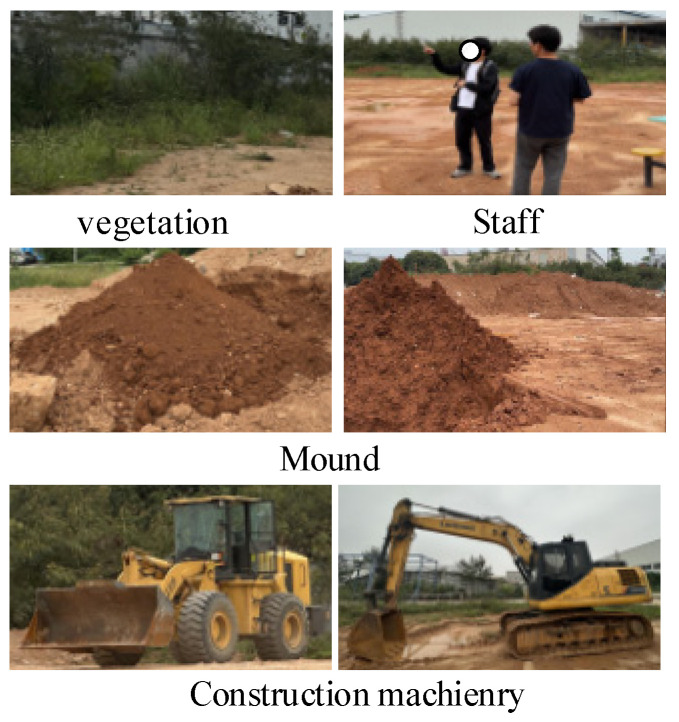
Data set target example.

Based on the actual working conditions of construction machinery, common objects in unstructured scenes—such as mounds, personnel, construction equipment, unstructured roads, vegetation, and shrubs—are selected as representative targets. Five categories of these objects are illustrated in Figure 5. The annotated multi-sensor semantic segmentation dataset for excavators in unstructured environments presented in this paper comprises 569 paired samples, establishing a foundational dataset for research and testing in excavator environment perception.

It should be emphasized that the core contribution of this work is not another fully supervised, data-intensive 3D perception model. Instead, a novel weakly supervised framework that entirely eliminates the need for 3D point cloud annotations is proposed, relying only on more accessible 2D image labels. The dataset constructed in this study primarily serves as a benchmark to validate the feasibility and effectiveness of the proposed fusion-and-optimization pipeline. Although smaller in scale compared to large-scale autonomous driving datasets such as KITTI, this limitation stems from the significant challenges and high costs associated with data acquisition and—more critically—the accurate annotation of 3D point clouds in unstructured environments. The chaotic and dynamic nature of such scenes makes manual point-wise semantic labeling exceptionally labor-intensive and prone to error.

### 2.2. Point Cloud Processing Branch

The point cloud branch constitutes the first parallel processing stream in our framework. Its objective is to segment raw 3D LiDAR data into distinct objects without relying on semantic annotations, thereby establishing a foundation for subsequent fusion. This section details the associated data processing and clustering procedures. While deep learning-based 3D point cloud segmentation typically depends on manually labeled data, unsupervised learning holds considerable potential in the field of autonomous driving [17], particularly for scene understanding tasks such as feature extraction, clustering, recognition, and the segmentation of various objects within a scene.

#### 2.2.1. Data Processing

**Figure 6:**
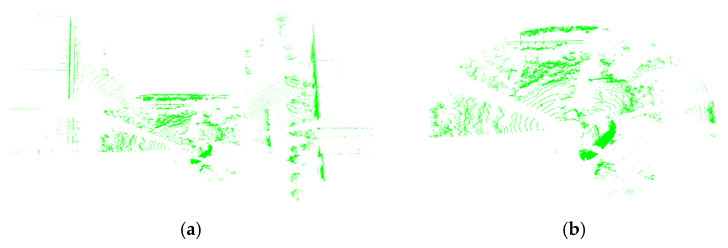
Comparison diagram before and after filtering. (**a**) Original point cloud data; (**b**) filtered point cloud data.

To meet the task requirements, the data undergo conditional filtering followed by ground point filtering. This study utilizes 128-line high-resolution LiDAR with a detection range of up to 220 m, generating approximately 240,000 points per scan frame. Filtering out point clouds beyond the effective operational area significantly reduces data volume. Given that the research focuses on a small electric excavator, whose low-speed operational radius is typically around 5 m, the effective working range is conservatively extended. A safety boundary of 40 m is established, and all data points beyond this radius are removed. This process reduces the point cloud count from 139,227 to 70,772, achieving a substantial decrease in data volume. A visual comparison of the point cloud before and after filtering is presented in Figure 6.

**Figure 7:**
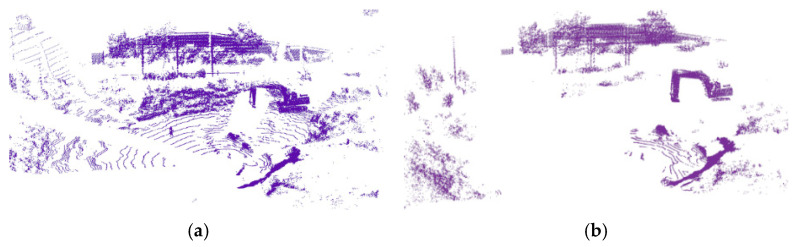
Point cloud ground point filtering based on RANSAC. (**a**) The ground point before filtering; (**b**) the ground point after filtering.

Point cloud data from unstructured scenes often contain a large number of disorganized ground points. Filtering these prior to processing helps reduce computational complexity [18]. Furthermore, for the semantic perception of the working facade during excavator operation, removing ground points allows target objects to exist independently within the data, thereby enhancing the performance and robustness of the subsequent clustering algorithm. The RANSAC algorithm [19] is employed for ground point filtering, which estimates the mathematical model parameters of the ground plane to identify and remove these points. The effect of this ground point filtering is shown in Figure 7.

#### 2.2.2. Clustering Model and Parameter Analysis

DBSCAN, which has a certain anti-noise ability [20], is selected as a point cloud branch algorithm to achieve the precise clustering segmentation task of the facade to be optimized. In the unstructured working environment of the excavator, the values of *MinPts* and *ε* are selected by the control variable method. According to the characteristics of the data, the minimum number of sample *MinPts* is set to 30, 40, and 50 as quantitative parameters, and *ε* is tested. The evaluation indexes of the DBSCAN clustering algorithm with different *ε* variables are given in in Table 4.

The selection of the clustering algorithm is critical for segmenting objects in unstructured scenes. DBSCAN is chosen over centroid-based algorithms like K-means for two primary reasons. In unstructured environments, the number of objects (clusters) in a scene is variable and unknown in advance. K-means requires the number of clusters k to be specified, which is impractical for our application. DBSCAN, in contrast, can discover an arbitrary number of clusters without this requirement. Objects like mounds and vegetation have highly irregular, non-spherical shapes and may be surrounded by noise points (e.g., sparse vegetation, dust). DBSCAN is capable of identifying clusters of arbitrary shape and is robust to noise, while K-means tends to produce spherical clusters and is sensitive to outliers.

The contour coefficient, Calinski–Harabasz (CH) index [21], and Davies–Bouldin (DB) score [22] are used as the evaluation indexes of clustering results. It can be found that with the increase of *ε* distance, there will be a better clustering effect, and the DB score reaches the minimum value when *ε* is 2.5 m. Further, the clustering segmentation algorithm is analyzed by the control variable *Minpts*. Different *Minpts* are set to test the clustering evaluation index. Table 5 shows the clustering quantitative evaluation index of different minimum sample numbers under fixed *ε*.

**Figure 8:**
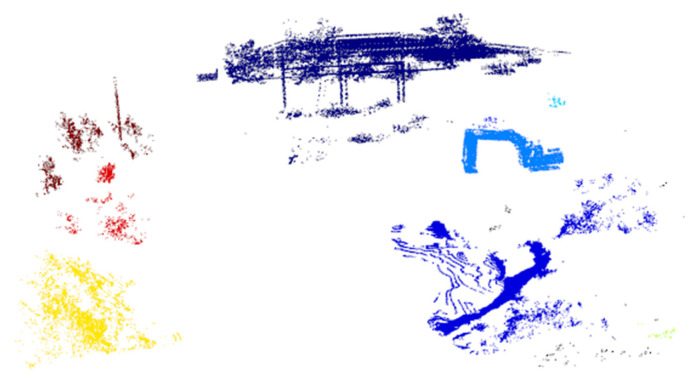
Clustering segmentation effect diagram of point cloud branch.

As can be found, when *Minpts* is 30, the DB score is the smallest. When *Minpts* is 100, the contour coefficient and CH index reach the maximum value. It is further found that when *ε* is 2.5 m, the clustering effect of most *Minpts* values meets the research task requirements. Based on the principle of selecting smaller clustering structures that help identify finer-grained clustering structures, requiring a smaller *Minpts* value to compensate for excessive noise, an *ε* of 2.5 m and an *Minpts* of 30 are selected as the parameters of the DBSCAN clustering algorithm. The clustering segmentation effect diagram of the point cloud branch is shown in Figure 8.

### 2.3. Image Semantic Segmentation Branch

Operating in parallel with the point cloud branch, the image branch is responsible for generating dense 2D semantic information. The output of this branch provides the semantic labels that will be transferred to the 3D point cloud. This section introduces the network architecture and training of our image semantic segmentation model. Based on the deep learning, the fully supervised learning codec structure is selected as the image semantic perception algorithm. The backbone network and loss function are determined.

#### 2.3.1. Splitting Network and Loss Function Design

**Figure 9:**
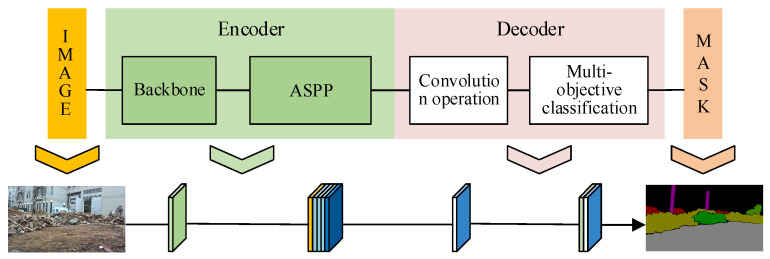
Image branch semantic segmentation network framework.

The selected image semantic segmentation network framework consists of an encoder and a decoder [23]. As shown in Figure 9, the encoder module is mainly composed of the feature extraction backbone network and the atrous spatial pyramid pooling (ASPP) module. The decoder part is used to scale the features of the encoder and restore the classification feature map with the same input resolution.

**Figure 10:**
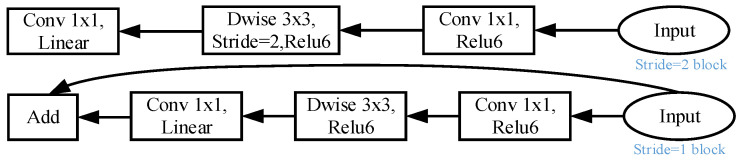
Backbone network inverse residual structure.

The backbone network is built by multiple inverse residual structures, namely inverted resblock. The inverse residual structure is based on the Residual structure in ResNet. The dimension-up and dimension-down modules are sequentially switched. Meanwhile, deep separable convolution is employed to perform point-by-point convolution on each channel. The output is combined to effectively reduce the number of parameters of the model. The inverse residual structure of the backbone network is given in Figure 10; the overall number of backbone network layers and the number of model parameters are lower, which means it can achieve a faster prediction speed.

**Figure 11:**
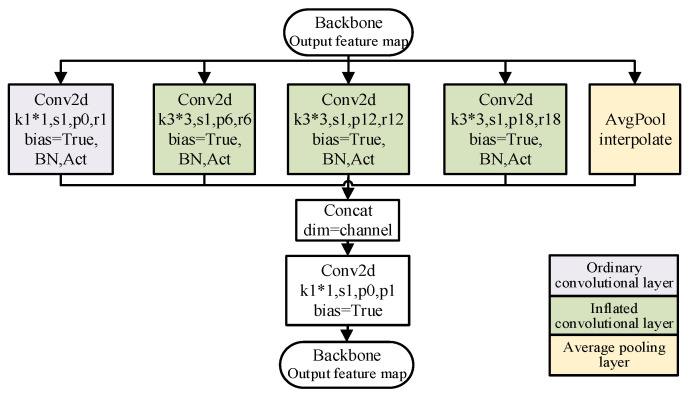
ASPP network structure.

The feature map after four convolution operations uses the expansion convolution of different expansion coefficients through the ASPP module feature extraction. The feature maps of different receptive fields output by different dilation convolution operations and the feature maps after pooling operations are superimposed and fused by adjusting the number of channels, and the high-dimensional semantic feature maps are output after dimension adjustment. The ASPP network structure diagram is shown in Figure 11. ASPP improves the recognition of different scale targets by different scale receptive fields, reduces the loss of information, and makes the network have a better segmentation effect.

**Figure 12:**
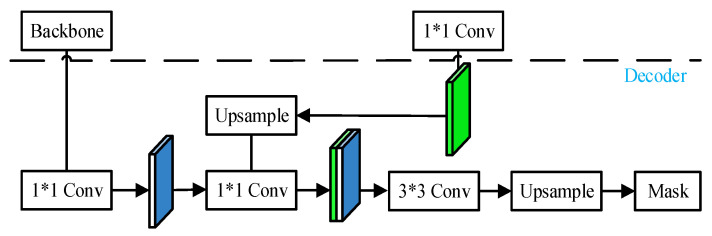
Decoding module network diagram.

In the decoding part, the 1 × 1 convolution operation is performed on the shallow features output directly from the trunk and the high-level semantic feature map after up-sampling. After adjusting the number of channels to the number of predicted categories, stacking fusion is performed. After the 3 × 3 convolution operation, the feature map of the same size as the original image is obtained. The semantic segmentation model is used to output the high-level semantics captured in the encoding phase as pixel-level mask prediction results, as shown in Figure 12.

Then, the combination of CE and Dice loss functions is selected as the network loss function for the semantic segmentation task of excavator facade obstacles in an unstructured environment. In the CE loss function [24], the amount of information is inversely proportional to the probability of the occurrence of the event, and the entropy is the expected value of all possible occurrences of the amount of information, which is the sum of the product of the results of the sum of the probabilities that may occur each time and can be obtained as follows:

(1) $$f(x)=\left\{\begin{array}{l} -\int p(X)\log p(X)dX,\;X\;\mathrm{is}\;\mathrm{continuous}\\ -\sum_{x}p\left(X\right)\log p(X),\;X\;\mathrm{is}\;\mathrm{discrete} \end{array}\right.$$

where $p\left(x\right)$ is the distribution probability of random variable X. The multi-classification cross-entropy loss function uses the One-hot vector to represent the true value $y_{ic}$ and can be expressed as follows:

(2) $$\mathrm{CE}(Y,\overset{\Lambda }{Y})=-\frac{1}{N}\sum_{i=1}^{N}\sum_{c=1}^{C}y_{ic}\log\left(\overset{\Lambda }{y_{ic}}\right)$$

where N is the number of samples, C is the number of categories, $y_{ic}$ is the probability of whether the $i_{th}$ sample is category C, and $\hat{Y}$ is the probability of the predicted value.

The Dice loss function [25] is calculated by evaluating the similarity between the binary masks of the two samples of the true value T and the predicted value P. The Dice coefficient is calculated as follows:

(3) $$\mathrm{Dice}(X,Y)=\frac{\left|X\cap Y\right|}{2\cdot \left|X\right|+\left|Y\right|}$$

In the task, there are only two real values of 0 and 1. The wrongly predicted sample points can be zeroed, and the wrongly predicted sample points can be punished. The predicted correct sample points obtain a higher Dice coefficient, thereby reducing the value of Dice loss. The Dice loss can be calculated as follows:

(4) $$\mathrm{DiceLoss}=1-\mathrm{Dice}=1-\frac{2\left|X\cap Y\right|}{\left|X\right|+\left|Y\right|}$$

where $\left|X\cap Y\right|$ is the intersection of the real label and the prediction mask. $\left|X\right|+\left|Y\right|$ is the union of the real label and the prediction mask. The smaller the Dice loss value, the better the prediction effect. In the case of multiple categories, each category is treated as an independent dichotomy problem to solve the Dice coefficient, and then the Dice coefficients of all categories are averaged to obtain the final loss function.

#### 2.3.2. Model Accuracy and Effect

**Figure 13:**
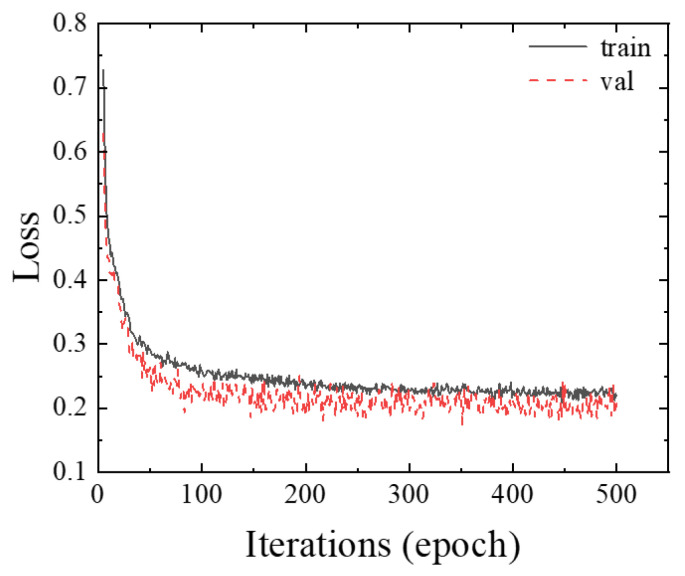
Model training loss curve.

For the constructed unstructured environment semantic data set, the pre-training model is used to train the unstructured environment semantic segmentation model through the transfer learning method. The batch_size of each batch sample is set to 4, and 500 epochs are trained, with a total of 71,000 iterations. As shown in Figure 13, the semantic data of the excavator unstructured environment are trained.

**Figure 14:**
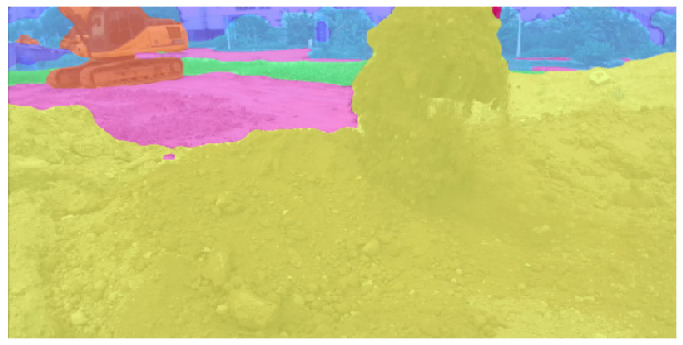
Prediction results of image semantic segmentation in an unstructured environment.

As can be seen, it tends to be stable after 500 epoch training. The loss curve is relatively stable. The loss value is low. This shows that it has a good training process, has strong generalization ability, and has no sign of fitting. The network model prediction and evaluation results are given in Table 6. It can be found that the network model shows better performance indicators in the unstructured environmental data of the excavator. The prediction results in an unstructured environment are shown in Figure 14.

### 2.4. Fusion Module

This section presents the core fusion and optimization module, which integrates the outputs from both preceding branches. The fusion process first projects the point cloud segmentation results onto the 2D image plane for semantic integration, after which the combined information is back-projected to generate initial 3D semantic labels. To address potential calibration errors, the perspective projection is refined using PSO. Subsequently, a Kd-tree-based Recurrent Neural Network (RNN) matching algorithm is applied to optimize the semantic and spatial consistency of the final 3D perception results.

#### 2.4.1. Fusion After Perspective Projection

This paper constructs the geometric mapping between point cloud clusters and image semantic pixels via a perspective projection model. A one-to-one correspondence is established through point-wise perspective transformation, mapping 3D point clouds onto a 2D plane with the same resolution as the image semantic data. One of the points $S_{3D}=(X,Y,Z)$ is taken in point cloud for study. Through the perspective projection matrix, the 3D space point is projected into the point on the focal plane of the camera and then mapped into the pixel plane point. The mapping calculation equation can be expressed as follows:

(5) $$\left\{\begin{array}{l} u=\frac{f_{x}X}{Z}+c_{x}\\ v=\frac{f_{y}Y}{Z}+c_{y} \end{array}\right.$$

where $f_{x}$ and $f_{y}$ are the focal lengths in x and y directions, respectively. $c_{x}$ and $c_{y}$ are the coordinates of the center point of the image. u and v are the pixel coordinates. X,Y and Z are the spatial coordinates.

To align the perspective projection of the point cloud with the image viewpoint, the transformation range is constrained by the image resolution, and points lying outside the image boundaries are filtered. For an image resolution of 1280 × 720 pixels, points with projected coordinates u<0, u>1279, v<0, or v>719 are discarded. Furthermore, as this study focuses on forward-looking perception, only point cloud clusters within the camera’s frontal field of view are retained. In the projection process, the depth value Z of a point denotes its distance to the image plane. Points with Z≤0 are excluded; Z=0 corresponds to a point coplanar with the observer (which is physically uncommon and introduces instability into the projection model), while Z<0 indicates a point located behind the camera. Therefore, only points with Z>0 are preserved for generating the 2D point cloud projection map.

**Figure 15:**
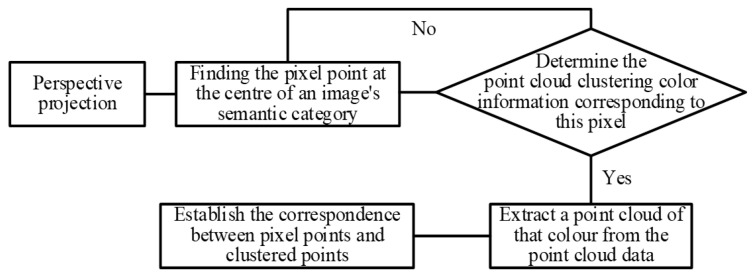
Establishment process of the corresponding relationship between point cloud point and pixel point.

Based on the initially calibrated extrinsic parameters, perspective projection is performed to align the point cloud cluster map with the pixel-wise semantic labels from the image. As illustrated in Figure 15, the image provides category-specific RGB values, while the point cloud clustering results only assign distinct color identifiers without semantic meaning. Objects such as construction machinery, personnel, and mounds, which exhibit clear spatial independence in clustering, serve as reference targets. Within a tolerable alignment error, the projected positions of most clusters overlap with their corresponding semantic regions in the image. By sampling the RGB values of several projected points near the centroid of each semantic area, a dictionary mapping cluster colors to semantic categories is constructed. This mapping allows the semantic labels from the image to be transferred to the corresponding 3D points. The annotated point cloud is then restored in 3D space, enabling the extraction of spatial contours for each category.

#### 2.4.2. Weak Calibration Optimization Based on PSO

**Figure 16:**
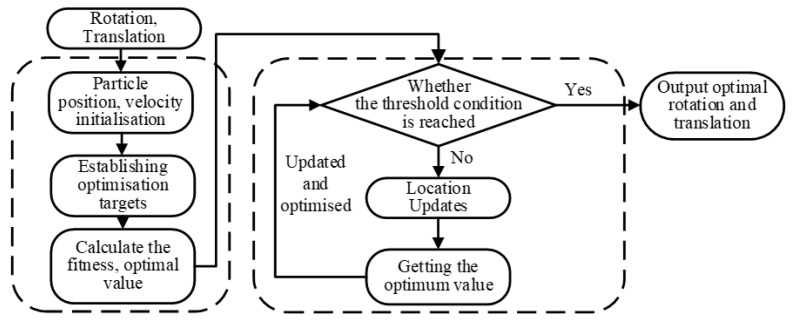
PSO flow chart.

Calibration errors in multi-sensor extrinsics can lead to misalignment during mapping and fusion. To address this, PSO is employed to refine the weakly calibrated parameters, enabling the perspective projection model to operate with optimal extrinsic relationships. Based on the established mapping between image categories and their corresponding point cloud clusters, the point cloud data for each target category, including construction machinery, personnel, and mounds, is extracted. The Intersection over Union (IoU) between these category-specific point clouds and the preliminarily fused semantic point cloud is defined as the fitness function. The six degrees of freedom in rotation and translation are encoded as particle positions. The PSO objective is to maximize this fitness. During iteration, each particle updates its velocity and position based on individual and group experience until convergence, yielding the optimal set of six extrinsic parameters. These optimized parameters are then applied to refine the extrinsic matrix, improving the alignment between the projected point cloud clusters and the image semantic segmentation results. The PSO workflow is illustrated in Figure 16.

**Figure 17:**
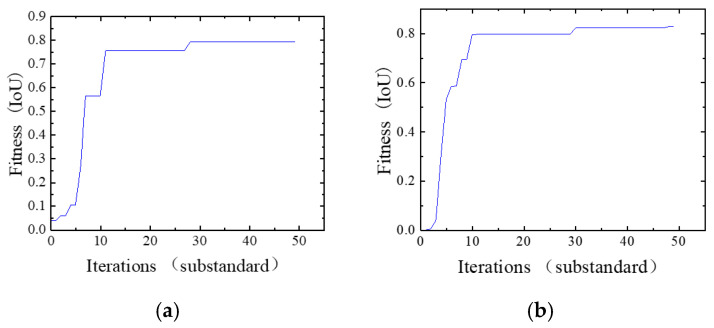

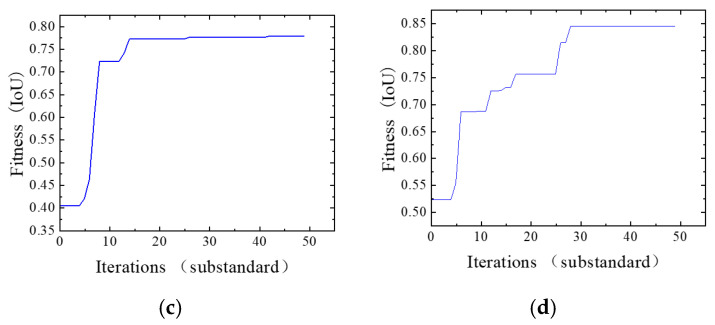
Sensitivity analysis of different particles in PSO. (**a**) Analysis curve of PSO when particle number is 3; (**b**) analysis curve of PSO when particle number is 6; (**c**) analysis curve of PSO when particle number is 12; (**d**) analysis curve of PSO when particle number is 18.

In this paper, three rotation values and three translation values need to be optimized through PSO. Therefore, the optimization dimension is set to 6. According to the data characteristics of the unstructured scene, the maximum range of the particle position is set to 0.6. The maximum velocity of the particle is generally 10% of the maximum range of the particle position, that is 0.06. If the number of iterations reaches 50 or the fitness value reaches 0.95, the optimization process is terminated. According to the empirical parameters, the particle swarm optimization is carried out for the four cases of particle number of 3, 6, 12 and 18. The corresponding relationship between the point cloud category and RGB value of the two categories of construction machinery and staff established above is used to establish the optimization objective function to optimize the external parameter relationship of particle swarm. As shown in Figure 17, it is the fitness of 50 iterations under four different particle numbers.

As shown in the results, when the particle number is set to 3, the fitness reaches approximately 0.77 around the 18th iteration, stabilizes after about 10 iterations, and attains a final fitness of 0.8055. With 6 particles, the fitness exceeds 0.8 by the 13th iteration, stabilizes shortly thereafter, and achieves a higher final fitness of 0.8253. For 12 particles, the fitness surpasses 0.7 by the 7th iteration, converges within 5 subsequent iterations, and results in a fitness of 0.7793. Using 18 particles leads to a fitness of 0.68 by the 7th iteration, followed by a stabilization period of nearly 20 iterations, yielding the best final fitness of 0.8461. Considering both convergence speed and final fitness, 12 particles are selected as the optimal configuration for balancing efficiency and performance. The PSO algorithm outputs a six-dimensional vector representing the refined LiDAR-to-camera transformation parameters, with the optimization performed using 12 particles (Table 7).

**Figure 18:**
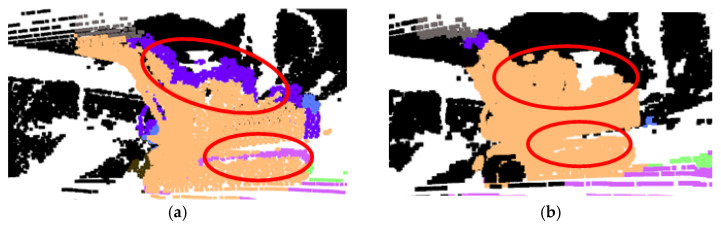

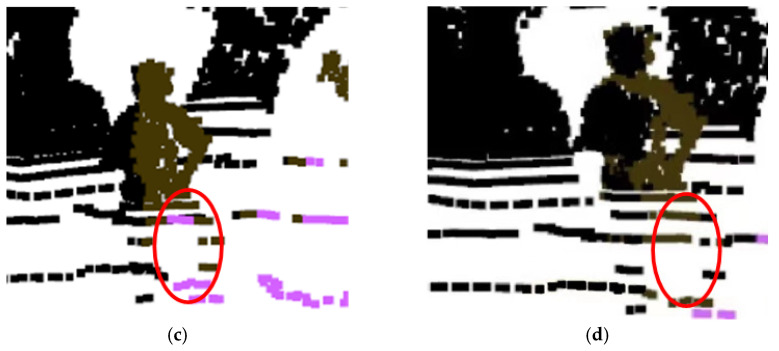
Comparison maps of the effect of the fused 3D semantic information before and after optimization. (**a**) Construction machinery before optimization; (**b**) construction machinery after optimization; (**c**) staff before optimization; (**d**) staff after optimization.

Using the extrinsic parameters optimized by PSO, the point cloud segmentation clusters are fused with the perspective-projected image semantic results. The fused semantic information is then restored into 3D space to output the final 3D semantic labels. Taking construction machinery and personnel as examples, Figure 18 presents a comparison of the 3D semantic perception results before and after optimization. The highlighted regions in the figure demonstrate that the accuracy of the fused semantic output is significantly enhanced after the optimized projection, with a notable reduction in fusion errors. While PSO improves geometric alignment, the semantic labels transferred to the point cloud may still contain inconsistencies. To address this, a final optimization step employing RNN-based matching is introduced to enhance semantic coherence.

#### 2.4.3. Adaptive RNN Optimization Based on Kd-Tree

This paper adopts an adaptive radius-based nearest neighbor (RNN) matching algorithm built on a Kd-tree structure [27] to enhance the semantic consistency of the fused 3D point cloud. A common challenge in nearest-neighbor methods is the manual and often arbitrary selection of the search range (e.g., *K* in K-NN or *R* in RNN). To address this, an adaptive strategy that determines an optimal search radius *R* for each object category, thereby improving robustness, is introduced.

**Figure 19:**
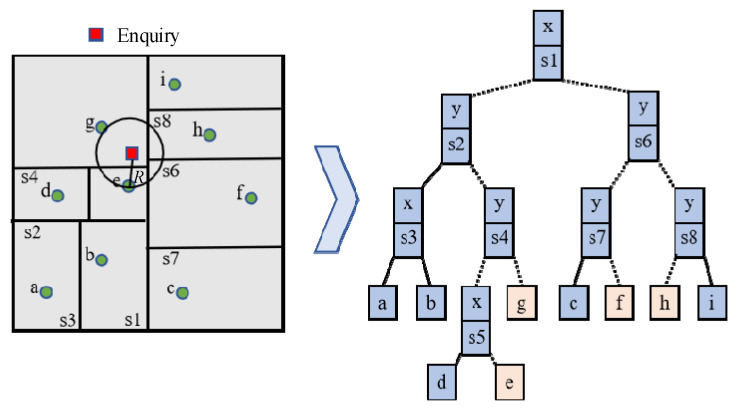
RNN search process.

The semantically fused point cloud is organized into a Kd-tree for efficient spatial indexing. As illustrated in Figure 19, the RNN search begins by inputting a query point *x* and its category-adaptive radius *R*. Starting from the root node, the algorithm recursively traverses the tree by comparing *x* with the splitting plane at each node until the containing leaf node is found, recording all points within *R* along the path. During backtracking, it checks whether the spatial region of each ancestor node intersects the query sphere. Only intersecting nodes and their subtrees are explored further; non-intersecting branches are pruned. This process continues until all relevant nodes are examined, ensuring an efficient and complete neighborhood query.

**Figure 20:**
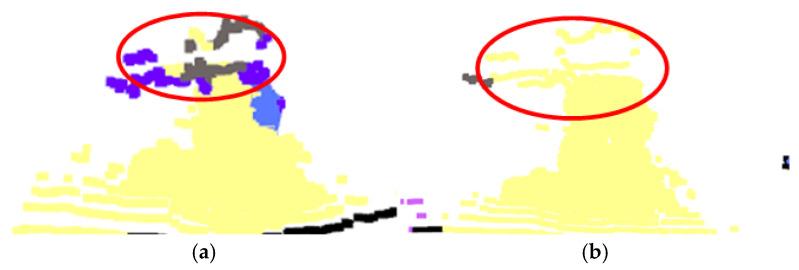
Comparison effect diagram before and after RNN optimization. (**a**) Before RNN optimization; (**b**) after RNN optimization.

The frequency of each semantic category among the neighboring points is recorded, and all points within the search radius of the query point are retrieved. The semantic label with the highest frequency is then assigned to the query point. By updating the labels of all points in this manner, the semantic consistency of the fused point cloud is enhanced, leading to improved segmentation accuracy. A comparison of the fused point cloud before and after nearest-neighbor matching is provided in Figure 20.

By updating the labels of all points in this manner, the semantic consistency of the fused point cloud is enhanced, leading to improved segmentation accuracy. This pipeline outputs a temporally consistent sequence of semantically labeled 3D point clouds, which forms the essential data foundation for dynamic 4D scene analysis (3D + time). Such 4D reconstruction and monitoring are recognized as valuable for progress management and constructability review in engineering projects, and they share technical parallels with spatio-temporal modeling in other fields [28].

## 3. Results and Discussion

### 3.1. Test Scene and Vehicle Platforms

**Figure 21:**
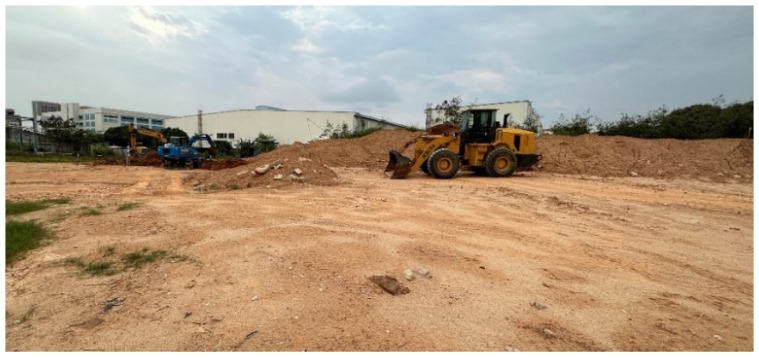
Test environment scene.

Figure 21 shows the test environment scene. The environment is a construction site where there is no fixed lane line, and the shape of the perceived object is irregular. The environment includes a variety of construction machinery, various shapes of earthwork to be operated, vegetation, staff, unstructured pavement and other target objects. It belongs to the typical unstructured working environment of an excavator.

**Figure 22:**
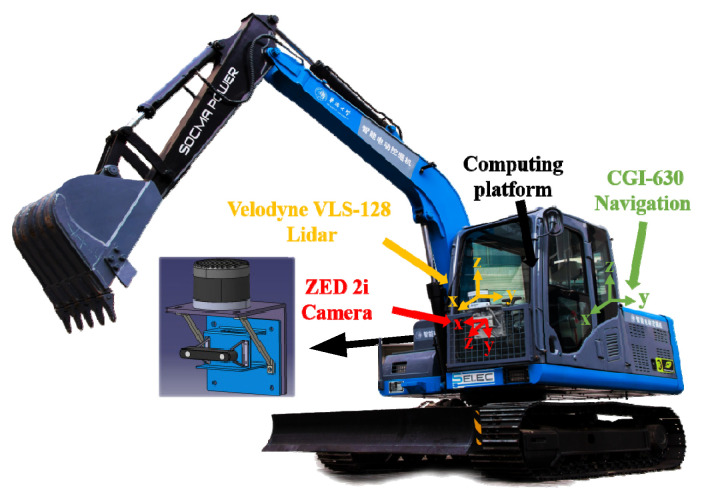
Vehicle test platform based on an electric excavator.

The experimental vehicle platform, based on an electric excavator, is shown in Figure 22. The excavator has a height of 2.6 m. A VLS-128 LiDAR is mounted on a sensor bracket at a height of 1.65 m, positioned 0.06 m to the right of the left track wheel center and 0.15 m behind the front track shaft center. A binocular camera is installed with its optical center at a height of 1.43 m and 0.02 m forward of the front track shaft, maintaining co-planar alignment with the LiDAR in the horizontal direction.

Given the computational cost of training and the consistency of results across the dataset, performance metrics in this study are derived from a single training run using a fixed train/validation/test split. The reported results thus represent a point estimate of the model’s performance on the designated test set.

### 3.2. Algorithm Test

Based on the aforementioned framework, the image branch employs the optimal parameter weights as the inference model for semantic prediction. The point cloud branch utilizes the DBSCAN clustering algorithm with parameters *ε* = 2.5 m and *MinPts* = 30 to segment the raw LiDAR data. The results from both branches are fused via perspective projection. Clusters corresponding to construction machinery and personnel—which exhibit clear spatial independence—are selected as optimization targets. A Particle Swarm Optimization (PSO) with 12 particles is applied to refine the 6-DoF extrinsic parameters (rotation and translation). The semantically fused point cloud is then restored to 3D space, and its consistency is further enhanced using a Kd-tree-based RNN matching algorithm. The semantic segmentation performance metrics for key façade targets—including mounds, personnel, construction machinery, and vegetation—are summarized in Table 8.

The experimental results demonstrate that the proposed algorithm achieves a segmentation accuracy and Intersection over Union (IoU) for construction machinery of 96.21% and 93.14%, respectively, indicating excellent performance for large, rigid objects, which served as a primary optimization target. For operational mounds, the accuracy and IoU reach 86.34% and 83.61%, also reflecting robust segmentation. In the case of personnel—dynamic and small targets—the accuracy remains high at 92.13%, but the IoU is 72.33%. This lower consistency primarily arises from the inherent sparsity of human-body point clouds and potential motion artifacts in dynamic scenes. The ground category, mapped using filtered ground points, attains an accuracy of 79.56% and an IoU of 70.87%. Vegetation, including shrubs with staggered distributions and weak feature similarity during clustering, shows weaker object independence, resulting in an accuracy of 69.38% and an IoU of 59.31%.

**Figure 23:**
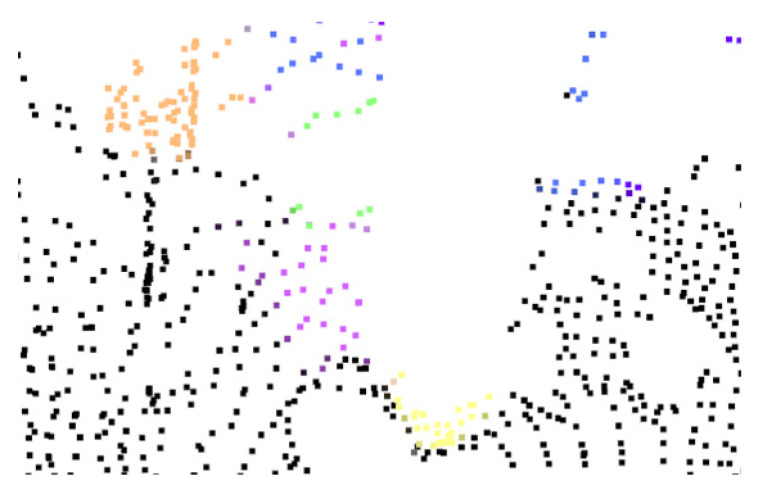
Output point cloud semantic segmentation results after sampling.

In summary, the proposed algorithm meets the semantic perception requirements for unstructured excavator environments, achieving an overall mean accuracy of 84.72% and a mean IoU of 75.85%. This performance is competitive for 3D semantic perception, which is particularly notable because it was attained using only 2D image labels during training, thereby substantially reducing annotation cost. The final sampled point cloud segmentation result (Figure 23) contains, within the perspective range, 84 points for construction machinery, 23 for personnel, 56 for operational mounds, 29 for vegetation, and 38 for the ground. It is important to emphasize that these results were achieved without any 3D point cloud semantic labels in training, highlighting the key advantage of our weakly supervised approach. Through quantitative metrics, segmentation visualization, and target contour reconstruction, the proposed multi-sensor fusion algorithm demonstrates robust semantic perception performance, especially for construction machinery and operational mounds.

### 3.3. Ablation Study

To further validate the effectiveness of the optimization strategies in the proposed multi-sensor post-fusion semantic perception algorithm, an ablation study was conducted. The study focuses on the following two aspects: the refinement of weakly calibrated perspective projection via the PSO algorithm and the enhancement of semantic consistency using the Kd-tree-based RNN algorithm. Under the condition that the image and point cloud branches produce consistent preliminary outputs, the following four configurations were tested: (1) no post-fusion optimization, (2) PSO optimization only, (3) RNN optimization only, and (4) combined PSO and RNN optimization. The corresponding results are summarized in Table 9. The findings demonstrate that the proposed post-fusion optimization strategies significantly improve the performance of 3D semantic segmentation.

To further evaluate the performance of the proposed multi-sensor post-fusion semantic perception algorithm, comparative tests were conducted against the following baseline methods: the image-based semantic segmentation model DeeplabV3Plus [23], the projection-based point cloud segmentation method RangeNet++ [29], and the point cloud network RandLA-Net [30] that uses random sampling. The performance comparison is summarized in Table 10. As shown in the table, our method achieves a competitive mIoU of 75.85%. Notably, it outperforms all fully supervised 3D segmentation methods that require point-cloud labels for training. This result highlights the following key advantage of our framework: by using only 2D image labels, our weakly supervised approach not only reduces annotation cost substantially, but it also attains comparable or even superior perception performance through effective multi-sensor fusion and optimization.

### 3.4. Analysis of Computational Efficiency and Deployment Feasibility

Although this framework integrates multiple modules, including image segmentation, point cloud clustering, projection optimization, and semantic consistency matching, its overall computational overhead remains manageable in practical deployment. The image branch leverages the lightweight MobileNetV2 backbone network (with only 23.5 million parameters), significantly reducing model complexity and inference time while maintaining high segmentation accuracy. This offers clear advantages over networks like Xception (219.9 million parameters). The point cloud branch employs unsupervised DBSCAN clustering, eliminating dependencies on extensive labeled data and complex neural network training. Optimal parameters (*ε* = 2.5 m, *MinPts* = 30) were experimentally determined and combined with Kd-tree data structures for efficient neighborhood search, further enhancing clustering efficiency. Within the fusion optimization module, PSO is employed for offline calibration optimization without introducing online perception latency. The Kd-tree-based radius nearest neighbor matching algorithm achieves O(log n) query efficiency on point cloud data, enabling rapid semantic consistency correction. Practical testing demonstrates that this framework operates stably with near-real-time performance on the NVIDIA AGX Orin embedded platform, validating its deployability in resource-constrained construction machinery environments. Future enhancements will focus on code optimization and hardware acceleration to further improve system operational efficiency.

## 4. Conclusions

This paper addresses the challenge of semantic perception in unstructured environments for construction machinery. Given the scarcity of relevant datasets and the high cost of acquiring 3D point cloud semantic labels in such settings, a multi-sensor fusion semantic segmentation algorithm is proposed, leveraging a binocular camera and LiDAR. The core of the approach lies in utilizing 2D image semantic labels to derive 3D semantic information, thereby circumventing the need for expensive point cloud annotations. To address imperfect sensor calibration during fusion, Particle Swarm Optimization (PSO) and a Recurrent Neural Network (RNN) are integrated to refine the extrinsic parameters. Validation in a real-world operational environment demonstrates that the proposed algorithm achieves competitive performance without relying on point cloud semantic labels, significantly reducing the cost associated with data annotation.

While the proposed weakly supervised 3D semantic perception framework achieves competitive performance using only 2D image labels, it has certain limitations. The method relies heavily on the accuracy of the 2D image segmentation branch, as any segmentation errors can propagate directly to the 3D results and are difficult to correct subsequently, especially under challenging conditions such as extreme lighting, severe occlusion, or unseen object categories. Additionally, the unsupervised DBSCAN clustering remains sensitive to parameter settings, which may require recalibration in significantly different environments. Nevertheless, this work establishes a practical, low-cost pathway for 3D scene understanding and provides a valuable foundation for future research.

## Figures and Tables

**Table 1:** Multi-sensor time synchronization error.

Type of Error	Average Error	Standard Deviation	Maximum Error	Minimum Error
Error time/s	0.008627	0.005176	0.04577	0.000073

**Table 2:** Space calibration results of camera and laser radar.

Camera and LiDAR external reference	Rotation	5.5921 × 10^−2^	−9.9801 × 10^−1^	3.0724 × 10^−2^
		−9.1031 × 10^−2^	−3.5754 × 10^−2^	−9.9521 × 10^−1^
		9.9433 × 10^−1^	5.2842 × 10^−2^	−9.2834 × 10^−2^
	Translation/m	1.0573 × 10^−1^	2.5184 × 10^−1^	5.9031 × 10^−2^
	Re-projection error	0.454 pixel (1280 × 720)		

**Table 3:** Configuration of the software platform.

Software Platform	Version Number	Software Platform	Version Number
Ubuntu	18.04	Cmake	3.15.3
ROS	Melodic	Open3D	0.17.0
CUDA	11.0	Sklearn	1.0.2
Python	3.8	Opencv	3.2.0
Pytorch	1.7.1		

**Table 4:** Evaluation indexes of the DBSCAN clustering algorithm with different *ε* values.

*Minpts*	*ε*/m	Contour Coefficient	CH Index	DB Score
30	1.5	−0.193341508	2668.750300	0.789566410
	2	−0.084197134	3360.311606	0.794287972
	2.5	0.034387844	5380.229222	0.581872494
	3	0.089010400	4531.160681	0.602314787
	4	0.310410960	6627.606777	0.581989097
	4.5	0.310671064	6656.869485	0.650747466
40	1.5	−0.049984972	2670.392358	0.645354991
	2	−0.089984090	3331.997625	0.805917805
	2.5	0.070584830	4449.248430	0.620790919
	3	0.084487582	6590.531495	0.666087526
	4	0.316986744	5403.477244	0.690981773
	4.5	0.317404996	5430.627241	0.683215887
50	1.5	0.157745243	3256.341448	0.751316065
	2	0.127620560	3786.926407	0.827085480
	2.5	0.170435736	4433.066944	0.688266660
	3	0.316909586	5376.360560	0.693408017
	4	0.316933425	5399.600930	0.692313066
	4.5	0.316996425	5403.771131	0.691096918

**Table 5:** Evaluation indexes of DBSCAN clustering algorithm with different *Minpts* values.

*Minpt*	ε/m	Contour Coefficient	CH Index	DB Score
10	2.5	0.081212162	3472.769772	0.660031437
20		0.058834873	3837.998915	0.651435441
30		0.034387844	3380.229222	0.581872494
40		0.070584830	4449.248430	0.660790919
50		0.170435736	4433.066944	0.688266660
60		0.170123735	4450.486768	0.758329825
70		0.126135561	3832.895495	0.787773468
80		0.171001767	4428.676485	0.793797567
90		0.308265621	6526.951642	0.849448307
100		0.308226464	6522.299587	0.849342423

**Table 6:** Network model prediction and evaluation results.

Backbone	Loss Function	MIoU/%	MPA/%	Accuracy/%
MobileNetV2 [26]	CE + Dice	79.19	83.97	97.97

**Table 7:** Optimized results by PSO.

		*X*-Axis	*Y*-Axis	*Z*-Axis
Results	Rotation/rad	3.5275 × 10^−2^	−9.6083 × 10^−3^	1.0868 × 10^−3^
	Translation/m	−1.2364 × 10^−1^	−3.9860 × 10^−2^	−1.9693 × 10^−2^

**Table 8:** Multi-sensor fusion semantic perception performance index.

Index	Staff	Construction Machinery	Mounds	Vegetation	Ground	mIoU/%	mPA/%
IoU/%	72.33	93.14	83.61	59.31	70.87	75.85	\
PA/%	92.13	96.21	86.34	69.38	79.56	\	84.72

**Table 9:** Ablation study.

Condition	PSO	RNN	mIoU/%	mPA/%
1			56.87	69.76
2		√	67.66	76.23
3	√		68.87	77.04
4	√	√	75.85	84.72

**Table 10:** Performance comparison of semantic segmentation algorithms.

Types	mPA/%	mIoU/%	Semantic Label	Perception Dimensions	Sensor
DeeplabV3Plus	83.94	79.25	Image	2D	Camera
RangeNet++	81.98	69.33	Point cloud	3D	LiDAR
RandLA-Net	85.78	74.53	Point cloud	3D	LiDar
Our	84.72	75.85	Image	3D	LiDAR + Camera

## Data Availability

The data of this study are available upon reasonable request.
